# Differential Cellular Responses to Class I and II Sphingomyelinase D: Unraveling the Mechanisms of *Loxosceles* Venom-Induced Dermonecrosis and Potential Therapeutic Targets

**DOI:** 10.3390/ijms26073012

**Published:** 2025-03-26

**Authors:** Bruna Fernandes Pinto, Priscila Hess Lopes, Carlos Eduardo Madureira Trufen, Ana Tung Ching Ching, Inácio de Loyola M. Junqueira de Azevedo, Milton Yutaka Nishiyama-Jr, Marcelo Medina de Souza, Paula C. Pohl, Denise V. Tambourgi

**Affiliations:** 1 Immunochemistry Laboratory, Butantan Institute, Avenue Vital Brasil, 1500, Butantã, São Paulo 05503-900, Brazil; b.pinto.proppg@proppg.butantan.gov.br (B.F.P.);; 2 PREVOR, Rue des Chasseurs-Ardennais 3, 4031 Liège, Belgium; 3 Laboratory of Applied Toxinology, Butantan Institute, Avenue Vital Brasil, 1500, Butantã, São Paulo 05503-900, Brazil; 4 Centre of Excellence in New Target Discovery, Butantan Institute, Avenue Vital Brasil, 1500, Butantã, São Paulo 05503-900, Brazil

**Keywords:** *Loxosceles* venom, sphingomyelinase D, dermonecrotic lesion, wound healing, substance P, VEGF-A

## Abstract

Dermonecrosis resulting from *Loxosceles* spider envenomation, primarily driven by the enzyme sphingomyelinase D (SMase D), is characterized by severe inflammation and nonhealing wounds. SMases can be classified as Class I or II based on their structural characteristics. Class I exhibits greater dermonecrotic activity than Class II; however, the intracellular mechanisms responsible for this difference remain poorly understood. The differential transcriptomics analysis of human keratinocytes treated with each toxin revealed that Class I primarily activates pathways associated with proteolytic activity and apoptosis. In contrast, Class II uniquely upregulates key genes, including PIM-1, MCL-1, PAI-1, p21, and c-FOS, which support cell survival and inhibit apoptosis. These pathways also facilitate tissue repair and keratinocyte proliferation during wound healing, particularly through signaling mechanisms involving Substance P and VEGF-A. RT-qPCR confirmed these findings, with protein level evaluations indicating the sustained upregulation of VEGF-A exclusively in keratinocytes treated with Class II. We identified Substance P and VEGF-A as potential therapeutic targets for managing cutaneous loxoscelism, providing valuable insights into the cellular mechanisms underlying the distinct toxic effects of the two SMase D isoforms. By elucidating these pathways, this study enhances our understanding of loxoscelism’s pathophysiology and highlights strategies for therapeutic intervention in dermonecrotic injuries caused by spider venom.

## 1. Introduction

Loxoscelism, the envenomation by spiders of the genus *Loxosceles* (Araneae, Sicariidae), represents a significant public health concern in Brazil [1,2]. This condition can lead to severe local and systemic reactions, posing serious health risks to affected individuals [3,4]. While systemic manifestations such as intravascular hemolysis, disseminated intravascular coagulation, and renal failure are less frequent, they can occur alongside more frequently observed local effects like extensive tissue destruction, chronic ulceration, and necrosis. These local manifestations characterize a clinical condition known as cutaneous loxoscelism (CL) [5,6].

Cutaneous loxoscelism manifests initially at the bite site, progressing through a cascade of localized pathological changes culminating in necrotic lesions. These lesions, the hallmark of CL, often lead to prolonged ulceration [5,7]. These pathological changes include edema, increased endothelial thickness, vasodilation, vessel wall degeneration, intradermal hemorrhage, dermis—epidermis dissociation, and the vacuolization of basal layer keratinocytes [8,9].

Studies have established that sphingomyelinase D (SMase D), a key *Loxosceles* venom component, underlies these diverse clinical manifestations and is central in the pathogenesis of loxoscelism [7,10,11]. SMase D acts on sphingomyelin (SM), a key lipid component of cell membranes, to produce ceramide, a bioactive lipid involved in cell signaling and membrane structure. Additionally, SMase D hydrolyzes lysophosphatidylcholine (LPC) to generate lysophosphatidic acid (LPA), another potent lipid mediator. Ceramide, derived from SM, plays roles in apoptosis and cell membrane integrity, while LPA, derived from LPC, modulates cellular functions such as platelet aggregation, endothelial permeability, and pro-inflammatory responses. These lipid mediators are central to the pathogenesis of loxoscelism, contributing to dermonecrosis and systemic complications [11,12,13,14,15].

We have successfully cloned and expressed the active functional isoforms of SMase D from *L. intermedia* (P1 and P2) and *L. laeta* (SMase I), demonstrating that these recombinant proteins fully replicate the biological activities of their native purified counterparts [12,13]. Based on structural aspects, the active isoforms of SMase D present in *Loxosceles* venoms have been classified into two groups: Class I includes SMase I from *L. laeta*, which has a single disulfide bridge and contains a variable loop; and Class II, which contains an additional intra-chain disulfide bridge linking a flexible loop to a catalytic loop, represented by the isoforms P1 and P2 from *L. intermedia* [12,13,14,15].

Our previous studies demonstrated that *L. laeta* venom, which expresses Class I SMase D, exhibits a greater potential to induce complement-dependent hemolysis and dermonecrosis when compared to *L. intermedia*, which contains only Class II SMase D in its venom [4,16,17]. Matrix metalloproteinases (MMPs), specifically MMP-2 and MMP-9, induced by Class II SMase D, have been implicated in CL development [18,19,20]. However, Class I SMase D not only induces the increased expression of MMP-2 and MMP-9 but also uniquely elevates the levels of MMP-7, an enzyme associated with keratinocyte death [18,19,20,21]. As our earlier studies have shown in animal models, SMase D also triggers neutrophil influx and the degradation of collagen fibers within the dermis [18], making it an important contributor to dermonecrosis following envenomation. Additionally, SMase D isoforms have been found to induce a reduction in epidermal growth factor receptor (EGFR) expression, contributing to dermonecrosis and hindering the wound healing process [18,19].

Despite advances in understanding the inflammatory pathways triggered by sphingomyelinases D [22], the specific molecular and signaling mechanisms responsible for the differential toxicity and dermonecrosis associated with Class I and Class II SMases D remain unclear. Therefore, this study aims to elucidate these mechanisms by investigating the distinct cellular responses elicited by each isoform, identifying potential therapeutic targets that could mitigate the pathological effects of cutaneous loxoscelism. Herein, we identified the potential involvement of Substance P and VEGF-A molecules in suppressing apoptosis and promoting tissue repair, highlighting their therapeutic potential in managing loxoscelism-induced injuries.

## 2. Results

### 2.1. SMase D Isoforms Induce Biological Processes Associated with Inflammation, While Class II Specifically Promotes Wound Healing Pathways

**Figure 1:**
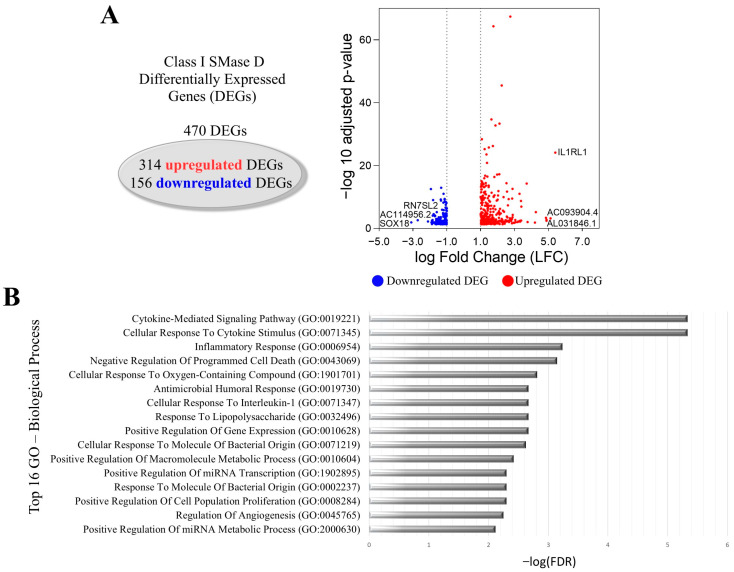
Transcriptional changes in keratinocytes after Class I SMase D treatment. (**A**) Volcano plot showing DEGs after 2 h treatment with Class I SMase D, highlighting the top 3 upregulated (red dot) and downregulated (blue dot) genes. (**B**) The top 16 biological processes associated with the LFC-ranked genes enriched by GSEA analysis using Gene Ontology (GO).

Through transcriptome analysis, we identified 470 differentially expressed genes (DEGs) in human keratinocytes treated with Class I SMase D. Among these, 156 genes were downregulated, while 314 were upregulated. Our key findings included the downregulation of *SOX18*, a transcription factor that plays a crucial role in various developmental processes and may influence the keratinocyte profile. Additionally, we observed the upregulation of *IL1RL1*, a gene that encodes a receptor involved in the inflammatory response, suggesting its role in mediating the keratinocytes’ response to cytokines. Using the Gene Ontology (GO) database, we identified the top 16 biological processes associated with these DEGs, including cytokine-mediated signaling, cellular response to cytokine stimulus, inflammatory response, cellular response to interleukin-1, and the positive regulation of cell population proliferation. These findings indicate that Class I SMase D significantly alters the gene expression profile of human keratinocytes, potentially influencing inflammatory responses and cellular proliferation (Figure 1A,B).

**Figure 2:**
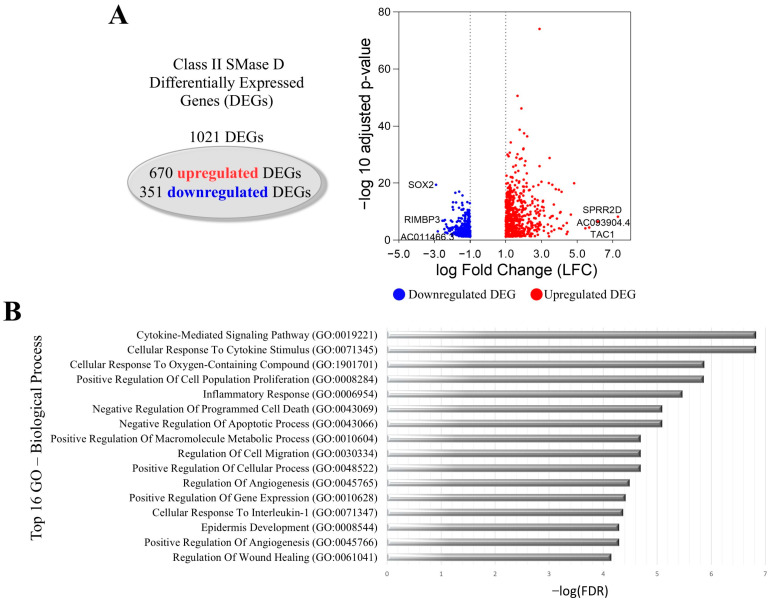
Transcriptional changes in keratinocytes after Class II SMase D treatment. (**A**) Volcano plot showing DEGs after 2 h treatment with Class II SMase D, highlighting the top 3 upregulated (red dot) and downregulated (blue dot) genes. (**B**) The top 16 biological processes associated with the LFC-ranked genes enriched by GSEA analysis using Gene Ontology (GO).

The treatment of keratinocytes with Class II SMase D resulted in 1021 DEGs, comprising 670 upregulated and 351 downregulated genes. Key findings included the downregulation of *SOX2* and the upregulation of *TAC1*, gene encoding the Substance P peptide, and *SPRR2D* (Figure 2A). GO analysis also identified the following among the top 16 biological processes associated with these DEGs: the cytokine-mediated signaling pathway, cellular response to cytokine stimulus, inflammatory response, and cellular response to interleukin-1. Additionally, we observed processes related to the positive regulation of cell population proliferation, the regulation of cell migration, epidermis development, and the regulation of wound healing (Figure 2B).

**Figure 3:**
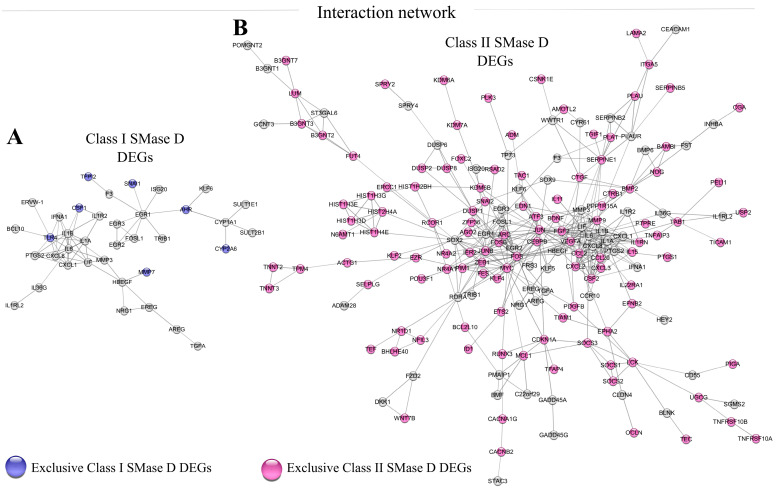
Network analysis of DEGs identified after Class I and Class II SMase D treatment. Interaction networks of DEGs identified after treatment with (**A**) Class I SMase D (470 DEGs) and (**B**) Class II SMase D (1021 DEGs) using String DB 12.0 and Cytoscape 3.10.3 software. Exclusive DEGs are highlighted in blue for Class I SMase D and pink for Class II SMase D. Only strong interactions (high confidence > 0.75) were considered.

Strong interaction analysis of the DEGs (high confidence > 0.75) using StringDB 12.0 and Cytoscape 3.10.3 revealed distinct clusters. Class I SMase D treatment showed a primary cluster associated with the inflammatory response, including interactions with the apoptosis-promoting gene *BCL-10* (Figure 3A). In contrast, despite Class II SMase D treatment also revealing a cluster related to the inflammatory response, this analysis additionally highlighted interactions with several genes involved in cell survival and apoptosis suppression, such as *PIM1*, *MCL1*, *CDKN1A*, and *BCL2L10* (Figure 3B). These findings suggest distinct regulatory mechanisms and cellular responses elicited by Class I and Class II SMase D treatments in keratinocytes.

### 2.2. Distinct Pathway Targets of Class I and Class II SMase D Treatments: Proteolysis and Apoptosis Versus IL-10 Signaling and Keratin Metabolism

**Figure 4:**
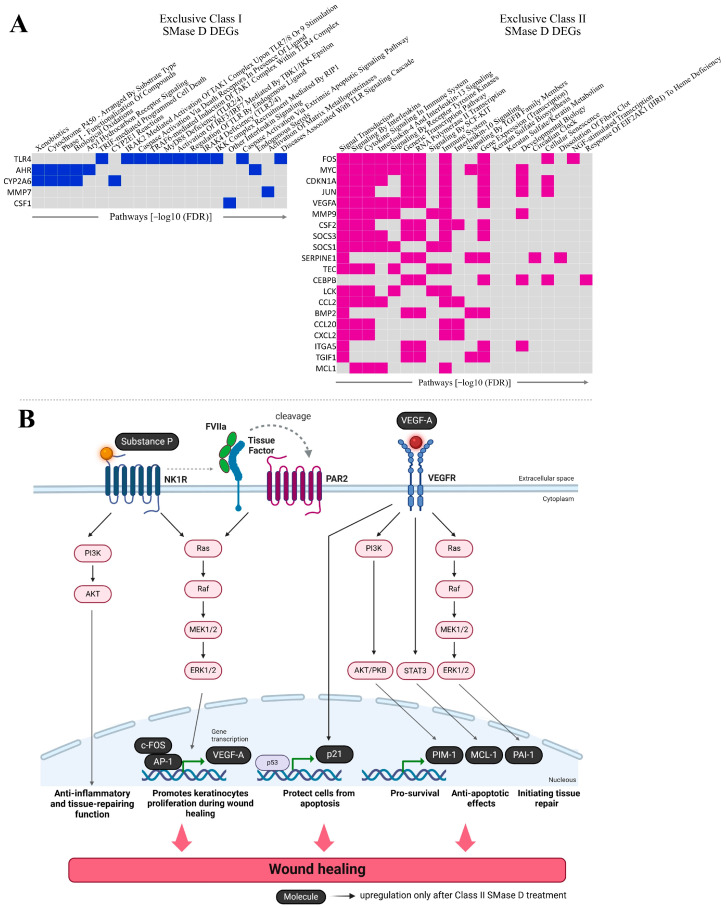
Functional enrichment and signaling pathway analysis of exclusive DEGs. (**A**) The top 20 signaling pathways enriched among the exclusive DEGs identified in the interaction analysis following treatment with Class I and Class II SMase D. Genes are ranked according to the number of pathways in which they are involved, as determined by EnrichR. (**B**) Schematic representation of the signaling pathways involving exclusive DEGs related to wound healing, identified only after treatment with Class II SMase D. Created with BioRender.com.

Further analysis of the exclusive DEGs clustered in Figure 3 following the treatment with Class I (highlighted in blue) or Class II (highlighted in pink) SMase D revealed 20 distinct pathways in which these exclusive DEGs are involved. The main pathways identified after Class I SMase D treatment included biological oxidation pathway, TRIF-mediated programmed cell death, IRAK2-mediated activation of the TAK1 complex upon TLR7/8 or 9 stimulation, caspase activation via death receptors in the presence of ligand, TRAF6-mediated induction of the TAK1 complex within TLR4 complex, the activation of IRF3/IRF7 mediated by TBK1/IKKε, other interleukin signaling, caspase activation via the extrinsic apoptotic signaling pathway, the activation of matrix metalloproteinases, and diseases associated with TLR signaling cascade. In contrast, the exclusive genes identified after Class II SMase D treatment were involved in the direct signaling by interleukins, cytokine signaling in immune system, interleukin-4 and interleukin-13 signaling, signaling by receptor tyrosine kinases, the immune system, interleukin-10 signaling, signaling by TGF-β family members, keratan sulfate biosynthesis, keratan sulfate/keratin metabolism, and developmental biology (Figure 4A).

Through transcriptome analysis, we identified the upregulation of *FOS*, *CDKN1A*, *VEGFA*, *SERPINE1*, *MCL1*, and *PIM1* genes exclusively following Class II SMase D treatment. These genes are involved in the regulation of the immune response, the suppression of apoptosis, tissue repair, and keratinocyte proliferation during wound healing. Based on these findings, we illustrate the potential cellular signaling pathways involving these genes, in conjunction with the action of *TAC1*, the most upregulated gene observed exclusively after Class II treatment (Figure 4B).

### 2.3. Distinct Temporal Expression Profiles Induced by Class I and Class II SMase D Treatments

**Figure 5:**
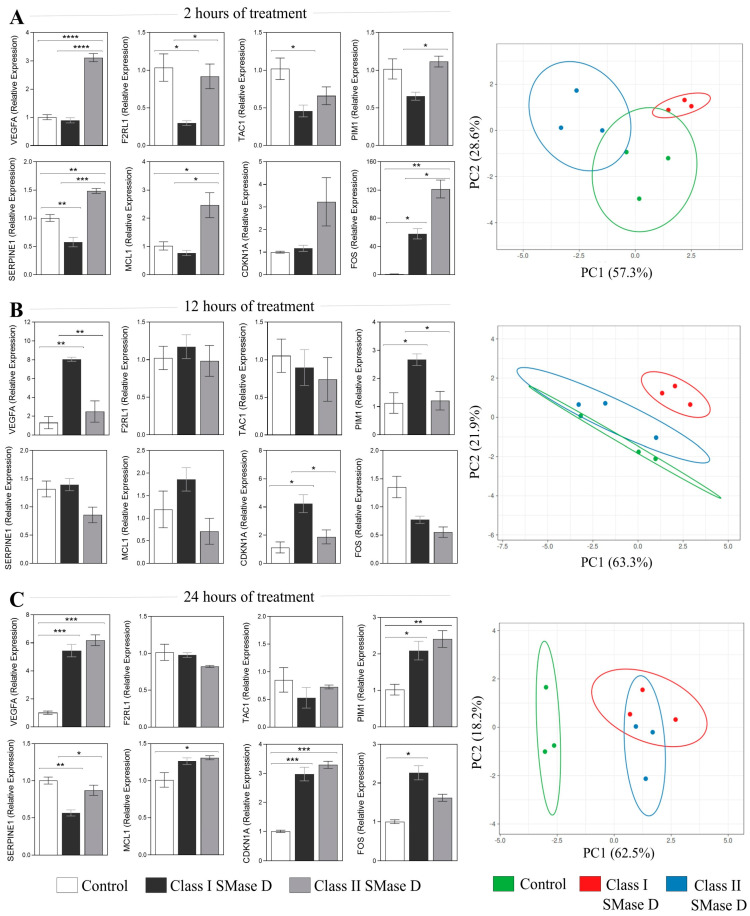
RT-qPCR validation of the key molecules after 2 h, 12 h, and 24 h of Class I and Class II SMase D treatment. Relative expression of the key molecules identified in the transcriptome analysis exclusively after Class II SMase D treatment at (**A**) 2 h, (**B**) 12 h, and (**C**) 24 h of Class I and Class II SMase D treatment. Principal component analysis (PCA) of the RT-qPCR data, generated using ClustVis 2.0 software. Prediction ellipses indicate a 0.95 probability that a new observation will fall within the ellipse. Statistical differences (*p* < 0.05) analyzed by one-way ANOVA followed by Tukey’s test are represented by asterisks, with *p* < 0.05 (*); *p* < 0.0021 (**); *p* < 0.001 (***); and *p* < 0.0001 (****). Data are represented as the mean ± standard error (SEM).

Our next step was to validate the expression of the aforementioned molecules using RT-qPCR following keratinocyte treatment with Class I or Class II SMase D for 2 h, 12 h, and 24 h. After 2 h of treatment, Class II SMase D induced a significantly higher relative expression of *VEGFA*, *SERPINE1*, *MCL1*, and *FOS* compared to both Class I SMase D treatment and the control. Additionally, *PIM1* and *F2RL1* also exhibited increased expression levels in response to Class II treatment relative to Class I. Conversely, Class I SMase D treatment resulted in lower relative expression levels of *TAC1*, *SERPINE1*, and *F2RL1* compared to the control. Notably, Class I treatment also led to a higher relative expression of *FOS* when compared to the control (Figure 5A). Principal component analysis (PCA) revealed distinct behavioral patterns between the two treatments and the control (Figure 5A).

At the 12 h mark, we observed a higher relative expression of *VEGFA, PIM1*, and *CDKN1A* exclusively after Class I treatment (Figure 5B). PCA indicated a higher distinct response observed following Class I SMase D treatment when compared with Class II SMase D treatment and the untreated control (Figure 5B).

At the 24 h time point, both SMase D treatments induced a similar modulation of *VEGFA*, *PIM1*, and *CDKN1A*. Additionally, higher expression levels of *SERPINE1* and *MCL1* were noted only after Class II SMase D treatment, along with a reduction in *SERPINE1* following Class I treatment compared to the control (Figure 5C). PCA further reinforced the distinct response observed following the SMase D treatments compared to the untreated control (Figure 5C).

### 2.4. Sustained VEGF-A Expression Observed Only in Keratinocytes Treated with Class II SMase D

**Figure 6:**
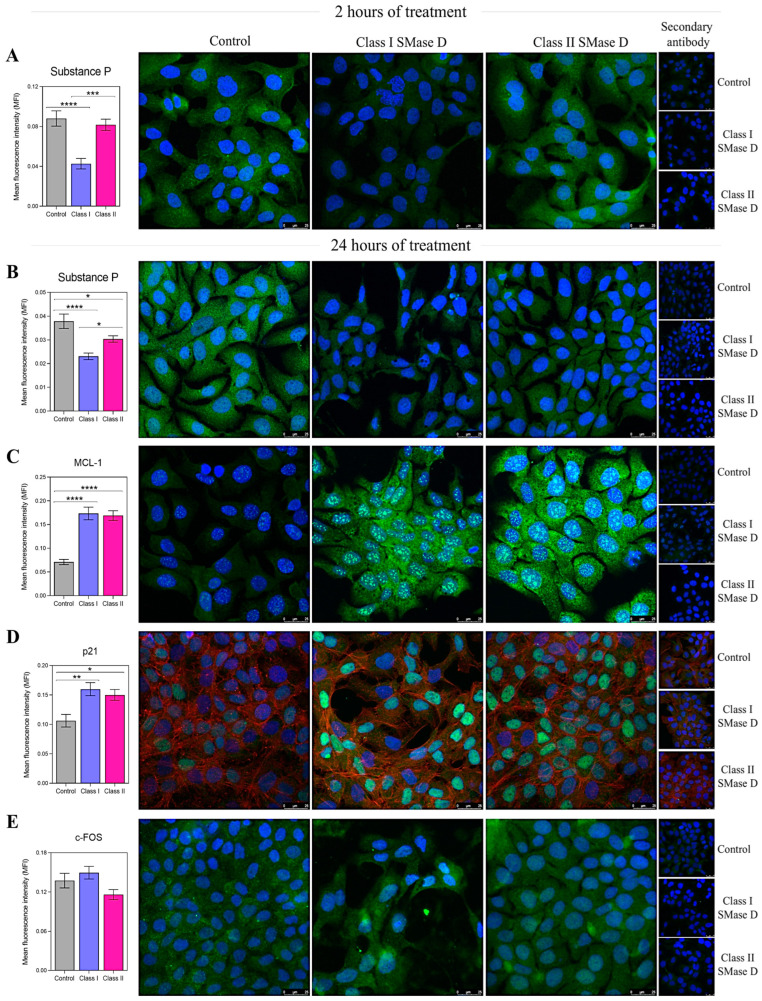
Immunofluorescence analysis of the key molecules after 2 h and 24 h of keratinocyte treatment with the control, or Class I or Class II SMase D. The mean fluorescence intensity (MFI) of (**A**) Substance P production in keratinocytes treated for 2 h with Class I or Class II SMase D; and (**B**) Substance P, (**C**) MCL-1, (**D**) p21, and (**E**) c-FOS production in keratinocytes treated for 24 h with serum-free DMEM, or Class I or Class II SMase D. Statistical differences (*p* < 0.05) were analyzed by one-way ANOVA followed by Tukey’s test for parametric data or Kruskal–Wallis followed by Dunn’s test for non-parametric data, and are represented by asterisks with *p* < 0.05 (*); *p* < 0.0021 (**); *p* < 0.001 (***); and *p* < 0.0001 (****). Data are represented as the mean ± standard error (SEM). Coverslips labeled only with secondary antibodies and unlabeled coverslips were used as technical controls. Cells were stained with the nuclear probe DAPI (blue) and FITC-labeled anti-rabbit secondary antibody to detect primary antibodies (green). For p21 labeling, we used phalloidin to detect F-actin (red). The scale bar represents 25 µm.

The next step was to evaluate the protein production of the targeted molecules using immunofluorescence at 2 h and 24 h post-treatment. After 2 h, we observed a higher relative expression of Substance P following treatment with Class II SMase D compared to Class I. Additionally, the expression of Substance P was lower after treatment with Class I SMase D compared to the control (Figure 6A). At 24 h, although we noted a reduction in Substance P production following both SMase D treatments compared to the control, cells treated with Class II SMase D still exhibited significantly higher levels of this molecule than those treated with Class I (Figure 6B). Furthermore, the increased production of MCL-1 and p21 was observed following treatment with both SMase D classes, further validating the results obtained through RT-qPCR (Figure 6C,D). However, no differences in c-FOS production were observed following the treatments at the analyzed time points (Figure 6E).

**Figure 7:**
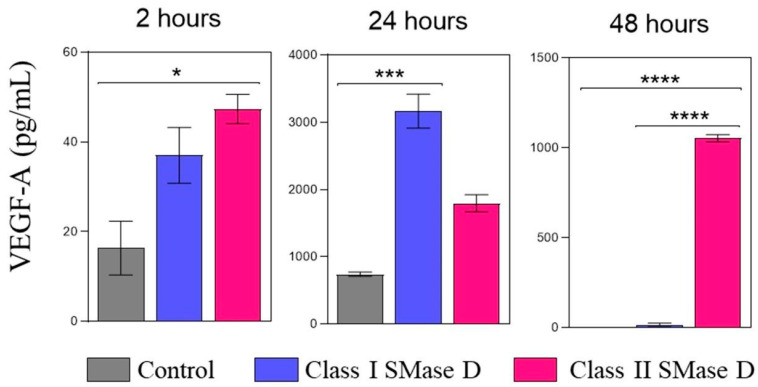
VEGF-A protein detection in the keratinocyte supernatant after 2 h, 24 h, and 48 h of Class I or Class II SMase D treatment. VEGF-A protein levels were measured by ELISA in human keratinocyte culture supernatants at 2 h, 24 h, and 48 h post-treatment with Class I or Class II SMase D, compared to the untreated control. Statistical differences (*p* < 0.05) were analyzed by one-way ANOVA followed by Tukey’s test for parametric data or Kruskal–Wallis followed by Dunn’s test for non-parametric data, and are represented by asterisks with *p* < 0.05 (*); *p* < 0.001 (***); and *p* < 0.0001 (****). Data are represented as the mean ± standard error (SEM).

We also assessed VEGF-A levels in keratinocyte culture supernatants after 2, 24, and 48 h of treatment with the control or Class I or II SMase D. At the 2 h mark, higher VEGF-A levels were detected following Class II SMase D treatment, corroborating the findings from our RT-qPCR and transcriptome analyses. After 24 h, the highest levels of VEGF-A were observed in the supernatants of keratinocytes treated with Class I SMase D. However, at the 48 h time point, higher VEGF-A levels were again noted exclusively after Class II SMase D treatment (Figure 7).

## 3. Discussion

SMase D leads to dermonecrosis, a well-established consequence of *Loxosceles* envenomation, and has been identified as the key enzyme in the venom responsible for the clinical manifestations observed in humans [5,7,11,18,22]. Cutaneous loxoscelism triggers the recruitment of polymorphonuclear cells and the activation of MMPs, as well as molecules involved in IL-1 and ErbB signaling pathways. These processes contribute to a persistent inflammatory response and exacerbate tissue damage, often resulting in wounds that may take several months to heal [18,22]. Notably, Class I SMase D from *L. laeta* exhibits higher dermonecrotic activity than Class II SMase D from *L. intermedia* [4,16,21]. However, the intracellular mechanisms underlying this contrast in envenomation effects between both SMase D classes remain unclear.

Transcriptome analysis revealed that the treatment with both SMase D classes directs biological processes and intracellular signaling related to the activation of inflammatory responses and cell proliferation. Interestingly, the most downregulated DEGs following Class I and Class II SMase D treatments were *SOX18* and *SOX2*, respectively. SOX2 induction in keratinocytes has been shown to promote cell migration and accelerate the wound healing process [23], while SOX18 may play a role in activating angiogenic molecules during healing [24]. Additionally, *IL1RL1* was the most upregulated DEG after Class I SMase D treatment. Based on these findings, we hypothesize that both SMase D classes induce an inflammatory response that delays cell migration and proliferation, which aligns with our previous observations [22].

Analysis of the exclusive DEGs revealed that Class I SMase D primarily activates pathways associated with proteolytic activity and apoptosis, highlighted by the *MMP7* and *BCL10* genes, with the latter linked to apoptotic pathways (Figure 3A). It is worth mentioning that MMPs play a critical role in triggering uncontrolled proteolytic tissue destruction, contributing to the development of chronic non-healing wounds [18,21,25]. In the context of cutaneous loxoscelism, previous studies have demonstrated that MMP-2 and MMP-9 expression is associated with the loss of cell viability [18,19,20,21]. Furthermore, previous research conducted by our team showed that MMP-7 expression is significantly increased exclusively following Class I SMase D treatment [21], corroborating the transcriptome data and suggesting its contribution to the higher cell death levels in the treatment with Class I SMase D in comparison to Class II. Elevated MMP-7 can lead to extracellular matrix degradation, resulting in the dissociation of skin collagen fibers and contributing to lesion development and keratinocyte death [21]. These findings point out the importance of understanding the distinct roles of MMPs in the pathophysiology of loxoscelism, particularly concerning the differential effects of Class I and Class II SMase D.

In contrast to the major detrimental effects of Class I SMase D, the Class II SMase D treatment exclusively induced biological processes related to epidermis development and wound healing regulation, as well as exclusive genes associated with inflammation regulation via IL-10 signaling and keratin metabolism. Notably, *TAC1*, encoding the neuropeptide Substance P, was the most upregulated DEG.

Substance P is an 11-amino-acid neuropeptide produced by various cell types, including keratinocytes [26]. It interacts with the neurokinin-1 receptor (NK1R), which is expressed in nerves, immune cells, and epithelial cells, facilitating tissue regeneration and repair [27]. This interaction exhibits anti-inflammatory properties by inducing interleukin-10 (IL-10) and downregulating tumor necrosis factor-alpha (TNF-α) [28,29]. Here, we demonstrated significantly higher protein levels of Substance P following 2 h and 24 h treatment with Class II SMase D compared to Class I. Furthermore, we observed increased VEGF-A expression at both the mRNA and protein levels after 2 h of Class II SMase D treatment, consistent with the transcriptome analysis. VEGF-A, a member of the vascular endothelial growth factor (VEGF) family, plays a crucial role in the angiogenesis process. Keratinocytes enhance VEGF-A expression during tissue repair, promoting new blood vessel formation and wound re-epithelialization [30]. In the context of loxoscelism, a study demonstrated elevated VEGF’s expression in human keratinocytes following 2 h of treatment with *L. deserta* venom, supporting a role for VEGF in the wound healing process after envenomation [31].

As illustrated by Figure 4B, both Substance P and VEGF-A are critical molecules involved in activating signaling pathways that promote wound healing. These findings suggest that, in addition to inducing an inflammatory response that leads to tissue damage and delayed wound healing, keratinocytes treated with Class II SMase D appear to trigger a compensatory response. This response activates Substance P and VEGF-A-mediated intracellular signaling pathways, aiming to restore the healing mechanisms impaired by envenomation. Additionally, transcriptome analysis revealed the Class II SMase D specifically upregulated Proto-oncogene 1 (*PIM1*), *SERPINE1*, myeloid cell leukemia 1 (*MCL1*), *FOS*, and *CDKN1A*, all of which can be activated by Substance P and/or VEGF-A signaling. These molecules are involved in apoptosis suppression [32,33,34,35], tissue repair [36,37], and keratinocyte proliferation during wound healing [38]. Additionally, RT-qPCR validated these findings, confirming the higher relative expression of *PIM1*, *SERPINE1*, *MCL1*, and *FOS* exclusively after a 2 h treatment with Class II SMase D.

PIM1, a member of the PIM serine/threonine kinase family, plays a crucial role in regulating cell survival, exhibiting anti-apoptotic effects [39,40]. It has been shown that VEGF-A signaling increases PIM1 expression levels [41,42], which, in keratinocytes, is associated with cell differentiation [43]. SERPINE1 encodes plasminogen activator inhibitor-1 (PAI-1), which is upregulated following keratinocyte injury [37] and can be activated by VEGF-mediated ERK1/2 signaling [44]. PAI-1 is proposed to play a role in stimulating keratinocyte migration, and the loss of its function may hinder cutaneous wound closure [37]. MCL-1, an anti-apoptotic Bcl-2 family member, may also regulate keratinocyte differentiation [32] and is upregulated by VEGF [45]. In addition, Substance P can induce the gene expression and secretion of VEGF [46], indirectly contributing to the activation of the aforesaid molecules. Therefore, we propose that the activation of these molecules, triggered by Substance P and/or VEGF-A, may be crucial in facilitating lesion closure after envenomation by *L. intermedia*, whose venom contains Class II SMase D. This way, such signaling activation may potentially explain the relatively milder dermonecrotic effects of Class II SMase D.

However, after 12 h post-treatment with the toxins, the Class I-treated keratinocytes showed a distinct response: a higher relative expression of *VEGF-A*, *PIM1*, and *CDKN1A* compared to the Class II toxin and control, as well as significantly increased VEGF-A detection in the supernatant. *CDKN1A* is the gene that encodes the p21 molecule, a key cell cycle regulator [35], that can protect keratinocytes from apoptosis [47] by enhancing AKT activation [35,48]. Therefore, we hypothesize that, over the course of treatment, keratinocytes treated with Class I SMase D attempt to reverse the failure in the healing process of the toxin-induced damage by increasing the expression of molecules involved in apoptosis inhibition and cell proliferation. However, this modulation is ultimately ineffective, as the inflammatory response induced by Class I SMase D is exacerbated, initiating at 12 h of treatment, as evidenced by our previous findings [22]. This exacerbation contributes to persistent inflammation and increased dermonecrotic activity, as previously shown by our group [16,21,22].

In contrast, Class II SMase D-treated keratinocytes exhibited a sustained upregulation of *VEGF-A* and Substance P over time, which may be more effective in promoting wound healing and suppressing apoptosis. Furthermore, the activation of molecules such as *PIM1*, *SERPINE1*, *MCL1*, and *FOS*, mediated by Substance P and/or VEGF-A signaling, could contribute to the relatively milder dermonecrotic effects caused by Class II SMase D compared to Class I. Additionally, within 24 h of treatment, although both toxins induced a significant increase in the expression and protein production of *MCL-1* and *p21* as well as a high relative expression of *PIM-1*, which exhibited a similar profile when compared to untreated control, only keratinocytes treated with Class II SMase D continued to produce the tissue-repair-related molecule PAI-1 and the sustained high VEGF-A levels at 48 h. Thus, even though both SMase D classes seem to induce a similar profile over time by promoting the expression of molecules related to cell survival and proliferation, keratinocytes treated with Class II SMase D are exclusive in maintaining a consistently high expression of VEGF-A, a crucial molecule for activating signaling pathways involved in wound healing, over time. These results suggest that, unlike Class I, Class II SMase D may be more effective in promoting tissue repair. Although both toxins induce inflammatory processes and tissue damage, Class II SMase D appears to trigger a more sustained activation of pro-healing pathways mediated by Substance P and VEGF-A, which may contribute to the milder dermonecrotic effects observed in *L. intermedia* envenomation.

In this study, we elucidated for the first time the cellular mechanisms that establish the differences in the toxicity induced by Class I and Class II SMase D from *Loxosceles* spider venom. We found that the presence of SMase D, regardless of the *Loxosceles* species, can initiate an inflammatory process and impair wound healing, as previously documented in our previous study [22]. However, Class II SMase D uniquely activates Substance P and VEGF-A-mediated signaling pathways in keratinocytes, promoting immune response regulation, apoptosis suppression, tissue repair, and cell proliferation involving molecules such as PIM-1, MCL-1, PAI-1, p21, and c-FOS. Additionally, the sustained expression of these molecules, particularly VEGF-A, significantly enhances wound healing efficiency, while keratinocytes treated with Class I SMase D fail to induce these beneficial cellular mechanisms and instead, exhibit increased expression of the proteolytic enzyme MMP-7. Together, these factors contribute to the exacerbation of inflammatory processes and result in more severe dermonecrotic lesions.

Overall, we elucidated the distinct cellular mechanisms underlying the differential toxicity of Class I and Class II SMase D. Our findings highlight the potential roles of key molecules, particularly Substance P and VEGF-A as novel therapeutic targets for cutaneous loxoscelism treatment.

## 4. Materials and Methods

### 4.1. Recombinant Sphingomyelinase D

The recombinant SMase D from *L. intermedia* and *L. laeta* venoms were cloned, expressed, and purified as previously described [12,13]. The protein concentration of the SMase D samples was measured by the Lowry method [49] and the purity was evaluated by 12% sodium dodecyl sulfate polyacrylamide gel electrophoresis (SDS-PAGE) under non-reducing conditions [50]. Permission to access genetic resources registered under number AEE9AEA 11/01/2018 was provided by the National System of Management of Genetic Heritage and Associated Traditional Knowledge.

### 4.2. Cell Culture

The human keratinocyte cell line (HaCaT) obtained from Banco de Células do Rio de Janeiro (BCRJ) was cultured in 75 cm^2^ flasks (Corning Inc., New York, NY, USA) in Dulbecco’s Modified Eagle Medium (DMEM) supplemented with 10% fetal bovine serum (FBS) and 1% penicillin-streptomycin. The cells were maintained at 37 °C in a humidified atmosphere containing 5% CO_2_.

### 4.3. RNA-Seq Analysis

#### 4.3.1. Cell Treatments

HaCaT cells were cultured overnight in a serum-free DMEM medium. The following day, the cells were trypsinized using ATV (0.2% Trypsin and 0.02% Versene), washed, and resuspended in a serum-free DMEM medium. Triplicate samples of the cell suspension (1 × 10^6^ cells/mL) were then incubated for 2 h in a serum-free DMEM medium, supplemented with VBS^++^ buffer (2.8 mM barbituric acid, 145.5 mM NaCl, 0.8 mM MgCl_2_, 0.3 mM CaCl_2_, 0.9 mM sodium barbital, pH 7.2), with either phosphate-buffered saline (PBS—control) or 5 µg of the recombinant SMase D from *L. laeta* or *L. intermedia* venoms (1:1). Incubation was conducted at 37 °C with gentle agitation. Following incubation, the cells were washed with serum-free DMEM medium (centrifuged at 1500× *g* rpm and 4 °C for 10 min) and subsequently prepared for RNA extraction.

#### 4.3.2. RNA Extraction from HaCaT Cultures

Total RNA was extracted in triplicate using Trizol (Invitrogen, Thermo Fisher Scientific Inc., Waltham, MA, USA), which was directly added to the keratinocyte tubes (1 × 10^6^ cells), following the manufacturer’s protocol. Purified RNA was visualized via agarose gel, quantified using a Nanodrop 2000c Spectrophotometer, and re-quantified using the Quant-iT™ RiboGreen^®^ RNA Assay Kit (Thermo Scientific, Thermo Fisher Scientific Inc., Waltham, MA, USA). RNA integrity was evaluated using the Agilent 2100 Bioanalyzer (RNA 6000 Nano LabChip, Agilent Technologies, Santa Clara, CA, USA), confirming that all samples had an RNA integrity number (RIN) greater than 9.

#### 4.3.3. Library Preparation and Sequencing

Complementary DNA (cDNA) libraries were prepared from messenger RNA (mRNA) extracted from 1 µg of total RNA, following the protocol outlined in the TruSeq RNA Sample Prep Kit V2 (Illumina, San Diego, CA, USA). In summary, mRNA was isolated using oligo-dT and subsequently purified. The mRNA was fragmented by heating at 94 °C for 4 min in a fragmentation buffer. Double-stranded cDNA was synthesized, end-repaired, and A-tailed. Sequencing adapters were ligated to the cDNA fragments following the manufacturer’s protocol. The cDNA fragments were enriched by 15 cycles of PCR amplification. Quality control of the library was assessed by analyzing the size distribution of the cDNA libraries using a 2100 Bioanalyzer with the DNA1000 assay (Agilent Technologies, Santa Clara, CA, USA), and an ABI StepOnePlus Real-Time PCR System was used for quantifying the sample library before sequencing (KAPA Library Quantification Kit Illumina^®^ Platforms, Kapa Biosystems, Roche, Wilmington, MA, USA). The cDNA libraries were pooled to a final concentration of 12 pM and sequenced on an Illumina HiSeq 1500 System (Illumina, San Diego, CA, USA) in Rapid Run mode, using a paired-end flow cell for 200 cycles of 2 × 101 bp. The PhiX Control v3 kit (Illumina, San Diego, CA, USA) was employed as a control for the Illumina sequencing runs.

#### 4.3.4. Pre-Processing and Cleaning

The raw sequencing reads were cleaned by trimming and removing the reads with low quality and contaminants using in-house scripts as a wrapper for Fastq-mcf (v.1.04) [51] and Bowtie2 (v.2.5.5) [52]. Sequences shorter than 40 bp and low complexity regions above 90% of the sequence length were removed, retaining only reads with regions that have a quality score of 25 or higher.

#### 4.3.5. Mapping, Assembly, and Quantification

The high-quality reads were mapped to the human genome GRCh38, release 90 (Ensembl), using the alignment tool STAR [53]. Transcript quantification was performed using the featureCounts function implemented by the Rsubread R package [54]. Transcripts with low counts (less than one transcript per million) were excluded to prevent bias in subsequent analyses.

#### 4.3.6. Analysis of Differentially Expressed and Modulated Genes

The transcript abundance estimation was normalized for batch effects using the RUVg function of the RUVSeq R package [55], and the normalized count matrix was used as input for differential expression analysis using the edgeR R package [56]. Briefly, normalization factors were calculated using calcNormFactors with the TMM method [57], robust methods for dispersion were estimated using estimateDisp [58,59], and gene-wise negative binomial generalized linear models were fit using glmFit [60]. Genes with an absolute log2 fold change (LFC) greater than 1 and a false discovery rate (FDR) below 0.05 were considered differentially expressed (DEGs) [61]. DEGs were classified as upregulated or downregulated based on positive or negative LFC values, respectively. Volcano plots generated with GraphPrism 8.0 were used to visualize the results, facilitating the rapid identification of DEGs in each experiment.

Biological processes identified after each SMase D treatment were analyzed by Gene Set Enrichment Analysis (GSEA) [62] using the Gene Ontology (GO) database [63]. For functional association network analysis, we employed the STRING database [64] and Cytoscape 3.10.3 software [65], considering only strong interactions (high confidence > 0.75). Enrichr [66] was used to analyze the signaling pathways induced by the exclusive DEGs identified following the treatments. In these analyses, a threshold of -log10 (FDR) greater than 1.3 was considered.

### 4.4. RT-qPCR

RT-qPCR was performed on keratinocytes treated with Class I or Class II SMase D (5 μg) for 2 h, 12 h, and 24 h. RNA extraction from the keratinocyte culture, cDNA synthesis, and qPCR were performed as described [22]. A complete list of primers is described in Table 1. Relative mRNA levels were assessed in duplicates using the 2−ΔΔCt method with Glyceraldehyde-3-phosphate dehydrogenase (GAPDH) and Ribosomal Protein L13A (RPL13A) as endogenous reference genes [67].

### 4.5. Immunofluorescence Assay and Analysis on the Confocal Microscope

HaCaT cells were grown on coverslips in 24-well plates at 1.5 × 10^5^/mL in DMEM medium with FBS. After 24 h, the medium was replaced with serum-free DMEM, and the cells remained at this condition for another 24 h. Subsequently, the cells were treated in triplicate with Class I or Class II SMase D (10 µg) or serum-free DMEM as a control and incubated for 2 h or 24 h. Cells were washed with PBS, fixed with 4% paraformaldehyde for 15 min, and permeabilized with Triton X-100 (500 µL) for 10 min. The cells were washed with PBS and blocked by incubation with PBS, 1% BSA, and 0.05% Tween for 30 min, followed by 20 min of incubation with 10 µg/mL y-Globulin (y-Globulins G4386, Sigma-Aldrich, San Luis, MO, USA). Afterward, the coverslips were incubated with primary antibodies to Substance P (PA5-106934, Invitrogen, Thermo Fisher Scientific Inc., Waltham, MA, USA, 1:100), MCL-1 (ab32087, Abcam, Cambridge, UK, 1:100), c-FOS (MA5-15055, Thermo Fisher Scientific, Thermo Fisher Scientific Inc., Waltham, MA, USA, 1:100), and p21 (MA5-14949, Thermo Fisher Scientific, Thermo Fisher Scientific Inc., Waltham, MA, USA, 1:50) overnight. Samples were washed with PBS containing 0.05% Tween and incubated with FITC-labeled anti-rabbit secondary antibody for 1 h (F0382, Sigma-Aldrich, San Luis, MO, USA,1:80). For F-actin labeling, we used phalloidin (A22287, Alexa Fluor™ 647 Phalloidin, Thermo Fisher Scientific, Thermo Fisher Scientific Inc., Waltham, MA, USA), incubating samples for 1 h. Finally, coverslips were mounted on slides with 1 drop of Fluoromount-G with DAPI (00-4959-52, eBioscience, Thermo Fisher Scientific Inc., Waltham, MA, USA) and sealed to prevent drying out. Image acquisition was performed on a Leica TCS SP8 confocal microscope (Leica Microsystems, Wetzlar, Hesse, Germany). Cells were scanned along the x, y, and z axes using a 63×/1.4 NA objective and laser excitation at 405, 488, and 638 nm with LAS X software 3.5.7.23225 (Leica Microsystems, Wetzlar, Hesse, Germany). In total, 10 fields were acquired per coverslip in triplicate. CellProfiler™ Version 4.2.5 (www.cellprofiler.org) was utilized to assess the mean fluorescence intensity (MFI) of each molecule following exposure to Class I and Class II SMase D, as well as in the control group. For this analysis, we subtracted the MFI data obtained with only the secondary antibody from the MFI data obtained with both primary and secondary antibody labeling. Coverslips labeled only with secondary antibodies and unlabeled coverslips were used as technical controls.

### 4.6. VEGF-A ELISA in Cell Supernatant

VEGF-A protein levels in keratinocyte supernatants after treatment with Class I and Class II SMase D at 2, 24, and 48 h post-treatment were assessed using the human VEGF-A ELISA system (BMS277-2, Invitrogen, Waltham, MA, USA) following the manufacturer’s protocol. Samples and standards were performed in duplicate, and each treatment was carried out in biological triplicate. Briefly, the pre-coated wells from the microplate were washed twice with approximately 400 μL of wash buffer per well. Next, supernatants (1:2 dilution) and the different concentrations of standards were added to the wells. The plates were incubated at room temperature for 2 h. Up next, the wells were washed and 100 μL of biotin-conjugated detection antibody was added to the wells and incubated for 1 h at room temperature. Following another wash, 100 μL of Streptavidin-horseradish peroxidase-labeled (HRP) was added to the wells and incubated for 1 h at room temperature. Finally, a colorimetric reaction was developed using 3,3′,5,5′-tetramethylbenzidine (TMB) substrate for about 30 min and then stopped with the stop solution provided by the kit. The reaction was analyzed using a spectrophotometer reader at a wavelength of 450 nm. The concentration of VEGF-A in the samples was calculated based on the standard curve of recombinant VEGF-A (15.6 to 1000.0 pg/mL).

### 4.7. Statistical Analysis

Statistical analyses were conducted using GraphPad Prism 8.0 (San Diego, CA, USA), with a 95% confidence interval applied to all tests and statistical significance set at *p* < 0.05. The normality of the data distribution was evaluated via the Shapiro–Wilk test [68]. One-way ANOVA followed by Tukey’s test was used for parametric data, and Kruskal–Wallis with Dunn’s test was used for non-parametric data. Principal component analysis (PCA) was performed on RT-qPCR data to evaluate the behavioral profile of key molecules identified by transcriptome analysis following the different treatments. In this analysis, prediction ellipses indicated a 0.95 probability that a new observation will fall within the ellipse [69].

## Figures and Tables

**Table 1:** Sequence of primers used in RT-qPCR validation.

Target	Primer	Sequence
*TAC1*	Fw	GACTGGTACGACAGCGACC
	Rv	AAAGAACTGCTGAGGCTTGG
*VEGFA*	Fw	CCCACTGAGGAGTCCAACATC
	Rv	CTGCATTCACATTTGTTGTGCTG
*F2RL1*	Fw	CAGTGGCACCATCCAAGGAAC
	Rv	TTCCAGTGAGGACAGATGCAGA
*FOS*	Fw	GCTGGCGTTGTGAAGACCAT
	Rv	GTTGGTCTGTCTCCGCTTGG
*MCL1*	Fw	GAAGGCGCTGGAGACCTTAC
	Rv	GTTACGCCGTCGCTGAAAAC
*PIM1*	Fw	TCGGTCTACTCAGGCATCCG
	Rv	CTCGAGTGCCATTAGGCAGC
*CDKN1A*	Fw	GATGTCCGTCAGAACCCATGC
	Rv	CGCCATTAGCGCATCACAGT
*SERPINE1*	Fw	TCCACAAATCAGACGGCAGC
	Rv	CGTAGTAATGGCCATCGGGC
*GAPDH*	Fw	CCCACTCCTCCACCTTTGAC
	Rv	CCACCACCCTGTTGCTGTAG
*RPL13A*	Fw	GTATGCTGCCCCACAAAACC
	Rv	CTTCAGACGCACGACCTTGA

## Data Availability

The datasets generated during and/or analyzed during the current study are available from the corresponding author on reasonable request.
